# Study protocol to investigate the efficacy of normalisation of Advance Care Planning (ACP) for people with chronic diseases in acute and community settings: a quasi-experimental design

**DOI:** 10.1186/s12913-019-4118-x

**Published:** 2019-05-04

**Authors:** Sarah Jeong, Tomiko Barrett, Se Ok Ohr, Peter Cleasby, Michael David, Sally Chan, Helen Fairlamb, Ryan Davey, Peter Saul

**Affiliations:** 1 School of Nursing and Midwifery, University of Newcastle, PO Box 127, Ourimbah, NSW 2258 Australia; 2 Department of Aged Care Services, Wyong Hospital, PO Box 4200, Lakehaven, NSW 2263 Australia; 3 Hunter New England Nursing and Midwifery Research Centre, James Fletcher Campus, Gate Cottage, 72 Watt St, Newcastle, NSW 2300 Australia; 4 Division of Aged, Subacute and Complex Care, PO Box 6088, Long Jetty, NSW 2261 Australia; 5 School of Medicine and Public Health, University of Newcastle, Callaghan, NSW 2308 Australia; 6 School of Nursing and Midwifery, University of Newcastle, Callaghan, NSW 2308 Australia; 7 Cumbria Partnership National Health Service Foundation Trust. E27 Ruskin Corridor, The Carleton Clinic, Cumwhinton Drive, Carlisle, CA1 3SX UK; 8 School of Nursing and Midwifery, University of Newcastle, PO Box 127, Ourimbah, NSW 2258 Australia; 9 Calvary Mater Hospital & Newcastle Private Hospital ICU, Organ and Tissue donation for the Hunter New England Local Health District , New Lambton Heights, Australia

**Keywords:** Advanced care planning, Clinical trial, Nursing, Protocol, Normalisation, Chronic disease, Hospital, Community

## Abstract

**Background:**

Advanced care planning (ACP) is a process that involves thinking about what medical care one would like should individuals be seriously ill and cannot communicate decisions about treatment for themselves. The literature indicates that ACP leads to increased satisfaction from both patients and healthcare professionals. Despite the well-known benefits of ACP, it is still underutilised in Australia.

**Methods:**

The aim of this study is to investigate the effects of normalising ACP in acute and community settings with the use of specially trained normalisation agents. This is a quasi-experimental study, involving 16 sites (8 intervention and 8 control) in two health districts in Australia. A minimum of total 288 participants will be recruited (144 intervention, 144 control). We will train four registered nurses as normalisation agents in the intervention sites, who will promote and facilitate ACP discussions with adult patients with chronic conditions in hospital and community settings. An audit of the prevalence of ACP and Advanced Care Directives (ACDs) will be conducted before and after the 6-month intervention period at the 16 sites to assess the effects of the ACP service delivered by these agents. We will also collect interview and survey data from patients and families who participate, and healthcare professionals who are involved in this service to capture their experiences with ACP.

**Discussion:**

This study will potentially contribute to better patient outcomes with their health care services. Completion of ACDs will allow patients to express their wishes for care and receive the care that they wish for, as well as ease their family from the burden of making difficult decisions. The study will contribute to development of a new best practice model to normalise ACP that is sustainable and transferable in the processes of: 1) initiation of conversation; 2) discussion of important issues; 3) documentation of the wishes; 4) storage of the documented wishes; and 5) access and execution of the documented wishes. The study will generate new evidence on the challenges, strategies and benefits of normalising ACP into practice in acute and community settings.

**Trial registration:**

This project has been approved by the Hunter New England Human Research Ethics Committee (Approval No. 17/12/13/4.16). It has also been retrospectively registered on 3 October 2018 with the Australian New Zealand Clinical Trials Registry (Trial ID: ACTRN12618001627246). This study will operate in accordance with the National Health and Medical Research Council’s National Statement on Ethical Conduct in Human Research (2007) and the CPMP/ICH Note for Guidance on Good Clinical Practice.

## Introduction

Chronic disease (e.g. cardiovascular, respiratory, diabetes, dementia) contributes to more than 70% of the disease burden in Australia and the burden of chronic disease is increased with the ageing population [1, 2]. Among the patients with chronic conditions, a total of 17,372 people died after 38,905 hospitalisations representing 24% of total 165,000 hospitalisations in New South Wales (NSW) in 2011/12 [3]. The majority (76%) were emergency admissions. Although about 42% of this sub-cohort died in hospital, only 4% of them received palliative care services [3]. Given the significant impacts of rapid increase of ageing population with chronic diseases on the health of the people themselves and the resources of the health care system, understanding the issues related to end of life (EOL) care and treatment options preferred by people with chronic diseases has become a priority for health care professionals [2, 4].

Advance care planning (ACP) is a process that involves thinking about what medical care one would like should individuals be seriously ill or injured and cannot make or communicate decisions about care or treatment for themselves [5]. An Advance Care Directive (ACD) can only be made by an adult with decision-making capacity. It is a written statement by those who can make medical decisions for themselves for the time when one is unable to make decisions. It should include what is important to the person such as values, life goals and preferred outcomes [5]. It is widely considered optimal if ACP happens earlier in life, when the person is still well and capable of making decisions [6]. The benefits of ACP are well documented in Australian context and worldwide. ACP improves the quality of EOL care, patient and family satisfaction, and reduces stress, anxiety and depression in surviving relatives [7–15].

Significant work has been done to promote ACP internationally. In NSW, Australia, many resources are available about ACP and ACDs including legally binding ACD forms [5], and policies (e.g. End-of-life care and Decision-making Guidelines, A National framework for Advance Care Directives, Using Advance Care Directives, Advance Planning for quality care at end of life: Action Plan 2013–2018). Despite the well-known benefits of ACP for people, and substantial work conducted to increase the uptake of ACP, both published research [16] and our study’s initial evidence identified the following persisted problems in acute and community healthcare settings.

1. Despite many resources available on ACP and ACDs, awareness of ACP is low and completion of ACD is very low in The Hunter New England (HNELHD) and Central Coast Local Health Districts (CCLHD). This low rate is regardless of cultural and ethnic backgrounds, and stemmed from a lack of understanding of the concept, the various terms and forms, being time consuming in the processes involved and the feeling ‘I don’t know how to do it’ [17].
2. Adult children are the most preferred substitute-decision-maker (SDM) to husband/wife, but the existing legal and ethical frameworks do not capture the current preference of people as SDMs [18].
3. The attitudes towards the end-of-life issues differ within the context and depending on individuals’ cultural and religious beliefs and values, and preferences for care [19].
4. Despite education and resources available for health care professionals (e.g. doctors and nurses), they feel unconfident and unprepared, and their commitment remains minimal [16, 20]
5. The challenges in ACP lie in the processes of: 1) initiation of conversation; 2) discussion of important issues; 3) documentation of the wishes; 4) storage of the documented wishes; and 5) access and execution of the documented wishes, and that an effective and consistent solution for increasing practice of ACP remains elusive.

The proposed intervention in this project is informed by existing evidence that complex ACP interventions have resulted in increased frequency of out-of-hospital and out-of-ICU care, increased adherence to patients’ preferences, and increased satisfaction with their health care experience [7]. We established and tested evidence from the literature and clinical experiences over the last 10 years, and concluded that normalisation of ACP by a designated person and using the patient’s own language is the optimal implementation strategy. However, this approach has only been implemented and tested by a few individual clinicians on an ad hoc basis. The proposed intervention is built on these previous findings and will provide us with an opportunity to test and evaluate this approach in a wider scale.

In this study we examine the effect of trained general (not specialised) registered nurses (RNs) to promote and normalise ACP for people with chronic conditions in hospital and community health settings. The targeted population in this project (people with chronic diseases) is aligned with targeted populations for special Advance Care Planning needs [6], which includes; Diabetes, Congestive Heart Failure, Coronary Artery Disease, Chronic Obstructive Pulmonary Disease, Hypertension, Chronic Kidney Disease, Cancer, frailty and Dementia.

## Methods/design

### Aims

The primary aim: To test the feasibility and effectiveness of normalisation of ACP for people with chronic diseases in acute and community settings.

The secondary aims are:

1. To identify the characteristics of the most effective setting and demographics for ACP implementation.
2. To investigate the nature and extent to which planning for future health care wishes are initiated, discussed and documented among people with chronic diseases, their family members, and RN ACP facilitators.
3. To identify the effect of ACP on patient/SDM satisfaction with their healthcare experience.
4. To identify factors that influence the implementation of ACP in hospital and community health settings.
5. To assess and estimate the financial impact of normalisation of ACP on our health care system.

### Theoretical framework for the study

This project proposes an innovative approach to address the above problems by promoting ‘ACP conversations’ as part of routine (normal) clinical practice, underpinned by Normalisation Process Theory (NPT). This theory is centred on the work of embedding and of sustaining practices within interaction chains [21]. NPT focuses on what people do in the process of implementation. The theory constructs four mechanisms that explain the social process in the implementation of material practices by specialised ‘agents’. These mechanisms are coherence, cognitive participation, collective action, and reflexive monitoring (Table 1) [21].

**Table 1:** Agents and for mechanisms for Normalisation Process Theory

Agents	Agents are individuals and/or groups who contribute to the processes that lead to implementation, embedding, and integration of new practice. For this study, agents are healthcare professionals (admitting Medical Officer, Registered Nurse, Social Workers and RN ACP facilitators). They use the NSW Health ‘ACP – Making your wishes known’ information to patients, to initiate, engage in discussion, and answer any questions.
Coherence	This involves what and how things should be done which starts with how it has been done and what we should do differently. The meaning of new practice needs to be learned, experienced and internalised by the agents. The agents in this study are provided with the opportunities to learn, experience and internalise.
Cognitive participation	When the process of coherence is internalised, the agents engage in a new practice across the context. The agents in this study are given 6 months to internalise and normalise ACP as a routine practice.
Collective action	The new practice is operated and enacted in practice. In this study, material and human resources, working relationships between agents, a degree of autonomy are closely monitored and enacted upon.
Reflexive monitoring	This is an evaluation of implementation process. The agents will engage in an evaluation of the implementation process that reflects cognitive participation and collective action. It involves both individual and group appraisal and reconfiguration of ACP practice to embed and sustain a new ACP practice.

### Design

**Fig. 1:**
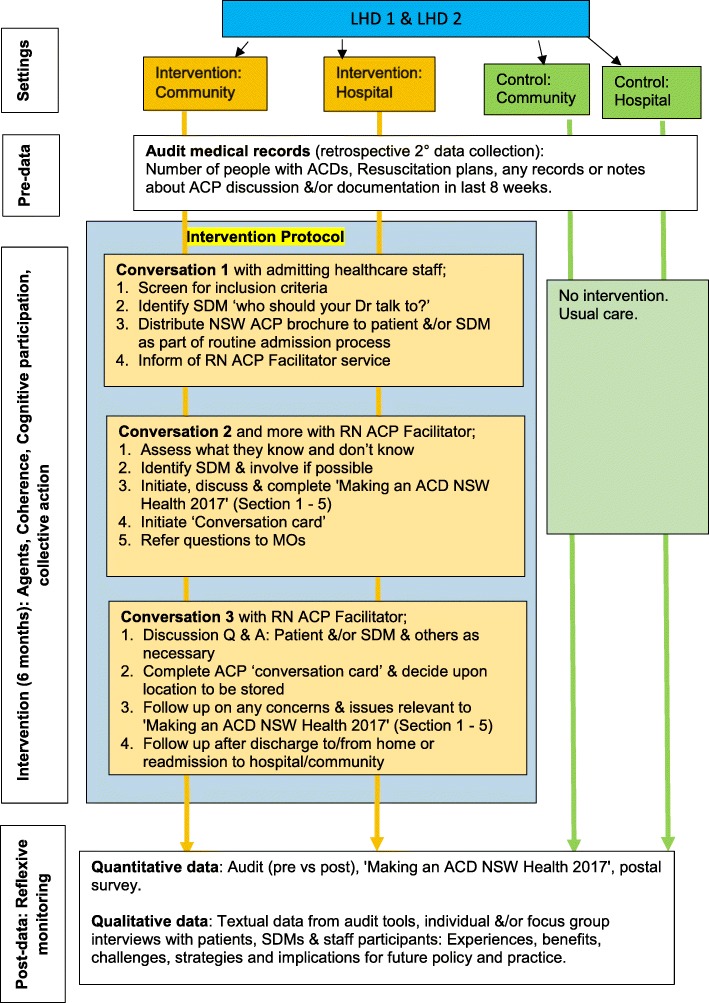
Research flow chart

The project is a quasi-experimental design with two groups: 1) intervention groups with RN ACP facilitators, and 2) control groups without RN ACP facilitators. See Fig. 1. Research Flow Chart below.

ACP will be normalised into practice for 6 months by RN ACP facilitators (normalisation agents) in nominated wards/units in acute and community settings in two Local Health Districts (LHDs). Pre and post measures (the number of people who have ACDs in medical records, Resuscitation plan, any records or notes about ACP discussion and documentation, and the concordance between the expressed wishes and the care delivered) will be obtained for the two groups. Post qualitative data (textual data, individual/focus group interviews with patients, SDMs and staff participants) will be collected and inform the challenges, strategies and implications for future policy and practice.

### Study setting and sample selection

Participants will be recruited from acute and community settings across LHD 1 and LHD 2, NSW, Australia. Research sites are listed below in Table 2.

**Table 2:** Research sites

	Acute		Community	
	Intervention	Control	Intervention	Control
LHD 1	Geriatric Rehab Unit (LHD1**HIS**1)	Geriatric Rehab Unit (LHD1**HCS**3)	Public Community Health Centre (LHD1**CIS**5)	Public Community Health Centre (LHD1**CCS**7)
	Medical ward at Hospital (LHD1**HIS**2)	Medical ward at Hospital (LHD1**HCS**4)	Non-public Home and Community Care (LHD1**CIS**6)	Non-public Home and Community Care (LHD1**CCS**8)
LHD 2	Medical ward at Hospital (LHD2**HIS**9)	Medical ward at Hospital (LHD2**HCS**11)	Public Community Health Centre (LHD2**CIS**13)	Public Community Health Centre (LHD2**CCS**15)
	Medical ward at Hospital (LHD2**HIS**10)	Medical ward at Hospital (LHD2**HCS**12)	Non-public Home and Community Care Service (LHD2**CIS**14)	Non-public Home and Community Care Service (LHD2**CCS**16)

Note: Hospital Intervention Site (*HIS*), Hospital Control Site (*HCS*), Community Intervention Site (*CIS*), Community Control Site (*CCS*). One full-time RN for 2 wards in one hospital and one full-time RN for 2 community service providers at one setting are allocated in each LHD

The 16 research sites were pair-matched (eight intervention, eight control) and selection was based on admission rates, patient profile, number of deaths per month/year, average length of stay and number of referrals from/to hospital and community. To minimise potential contamination of intervention the sites chosen are geographically separated. Both public funded and non-public sites are involved in this research, to maximise the generalisability.

All newly admitted patients to a site listed as an ‘intervention site’ will be offered facilitated ACP conversations, and all other sites will continue to deliver usual care (control sites).

Once potential participants are identified by inclusion and exclusion criteria (Table 3), a robust screening process will be applied which includes; patients with mental capacity and the ability to give valid informed consent as established by admitting medical officer or admitting RN by using Montreal Cognitive Assessment (MOCA) and Mini-Mental State Examination (MMSE). MOCA (26/30) and MMSE are done as part of new admission process (if a contemporaneous assessment has not already been completed by another unit/ward/department i.e. ED). Those who have a score of 10 or above will be nominated to participate. Those who have score of 10–24 (MOCA) and whose capacity is in question will be formally assessed by RN ACP Facilitators using NSW Capacity Tool Kit [22]. A conservative approach to recruitment will be applied. If capacity to consent is unclear for any potential participants these individuals will not be approached. Screening for potential participants will occur before consent has been obtained. This will prevent patients that are not defined as relevant to the aims of this research through the inclusion/exclusion criteria from being informed of a service they will not receive and/or overburdening people.

**Table 3:** Inclusion and exclusion criteria for participants in this study

Group	Inclusion criteria	Exclusion criteria
Group 1: People with Chronic Diseases	▪ Patients aged ≥18 years old ▪ Admitted to the wards/community centres in participating hospitals and community settings, ▪ Identified in Medical Records as having a chronic health condition(s) (defined within this research project as; Diabetes, Congestive Heart Failure, Coronary Artery Disease, Chronic Obstructive Pulmonary Disease, Hypertension, Chronic Kidney Disease, Cancer, Frailty and Dementia. ▪ Patients who do not currently have an Advance Care Directive	▪ Women who are pregnant and the human foetus. ▪ Children and/or young people (< 18 years old). ▪ People highly dependent on medical care. ▪ People who are experiencing acute severe physical illness and/or an acute episode of mental illness (a diagnosis of anxiety alone may not exclude participation)
Group 2: Substitute Decision Makers (SDMs)	• ≥18 years old • Nominated as an SDM by people with chronic diseases at intervention site • The ability to give valid informed consent	▪ Children and/or young people (< 18 years old). ▪ People who are experiencing acute severe physical illness and/or an acute episode of mental illness (a diagnosis of anxiety alone may not exclude participation)
Group 3: Admitting Healthcare Professionals (MOs, RNs, &/or SW) at intervention sites (normalisation agents level one, or NA1)	▪ ≥18 years old ▪ The ability to give valid informed consent ▪ Working in a professional capacity admitting patients to one of the research units listed, or ▪ Employed and trained as a NA2 for this research study.	▪ Not working in one of the research units listed
Group 4: RNs delivering the intervention (referred to as ‘RN ACP Facilitators’) at intervention sites (normalisation agents level two, or NA2).	▪ ≥18 years old ▪ The ability to give valid informed consent ▪ Employed and trained as a NA2 for this research study.	▪ Not employed and trained as a NA2 for this research study

### Sample size calculation

Power calculations were conducted to determine the sample size required to detect a change of at least 10% in the primary outcome; that being the proportion of completed NSW ACDs. Change was defined as the difference between the proportion at baseline, assumed to be 5% and at follow-up. Setting alpha at 5%, power at 80% and assuming a non-response rate of 25%, a sample size of 288 is needed (144 in intervention sites and 144 in control sites). Given an average admittance rate of at least 10 per week per site and over a six-month period, there is almost complete surety that the study will not be under-powered.

### Intervention

The intervention, ACP, is offered as part of routine service to the patients who are admitted to participating intervention wards/community centres. The intervention is a series of facilitated conversations about the components of ACP between people with chronic diseases, their SDMs and RN ACP facilitators.

#### Conversation 1

On admission, as a routine service, people with chronic diseases (and/or family if present) in intervention wards/community centres will be given a one-page ACP Brochure (produced by NSW Ministry of Health - Making your wishes known) by admitting Medical Officer (MO) &/or admitting Registered Nurse (and Social Worker if applicable).

#### Conversation 2 and more

All people with chronic diseases and their nominated SDMs who meet the inclusion criteria will be assessed by RN ACP facilitators for English Proficiency, cognitive impairment and acute episode of mental illness by asking the following questions as recommended by NSW Planning Ahead Tools [5].

1. Do you remember and/or understand the ACP brochure given by MO or RN or SW?,
2. Who should your doctor talk to about your medical treatment?
3. You have a choice to have or not to have a conversation(s) with me. Do you want to have a conversation(s) with me?

RN ACP facilitators will begin with open ended questions exploring the person’s knowledge, attitude and desire to participate in ACP. RN ACP facilitators will also clarify the person’s goals and values; identification of whom should be involved in these conversations; and the person’s understanding of diagnosis, prognosis and preferences for treatment options and place of care.

According to responses, RN ACP facilitator will initiate and facilitate a series of conversations between person, the nominated SDM, treating medical team, and/or a Health Care Interpreter or appropriate Cultural Support Person if required. The components of ACP include personal details, Person Responsible, personal values about dying, directions about medical care, specific requests for organ and tissue donation, and authorisation with signatures in Section 1 to 5 in NSW Ministry of Health ‘Making an ACD’ [5]. A summary of outcomes of these conversations will be entered: 1) in the person’s medical record; 2) A Conversation Log; and 3) A ‘Conversation card’ which is a size of business card when folded and which will be carried in participating patient’s wallet/purse.

Ongoing commitment from participants is not required. The series of conversations are optional and consent to proceed will be checked repeatedly through the course of discussion. The post-evaluative surveys and interviews are also voluntary and are ‘one-off’ so do not involve ongoing follow up or appointment. All potential participants will have as much time as they require to consider participation, but at least 24 h between provision of information (written information statement and verbal explanation) and gaining written informed consent for participation is recommended in line with the National Statement on Ethical Conduct in Human Research [23] and Good Clinical Practice guidelines [24].

### Data collection

#### Audit of ACP related practice

Data on the prevalence of ACP and ACDs will be collected through an audit of the medical records of patients admitted to the 16 research sites within the following time frames:

- The two month period before the introduction of the intervention period,
- The six month period of the intervention

The audit will initially check for the presence or absence of evidence of ACP only. Then the individuals whose medical records contain evidence of ACP will be invited to consent to access the content of ACDs (or documentation detailing personal values or specific wishes relating to medical care &/or organ donation). The audit involves the following steps.

1. Medical records from all individuals admitted to a research site at the given times will be requested from relevant medical records departments. Records will be audited for the presence or absence of ACP only.
2. Medical Record Numbers (MRN) of those containing evidence of ACP will be recorded. They will then be posted a letter of invitation and consent form with a pre-paid, self-addressed envelope.
3. The individuals will return the consent form directly to the Research team. Where a completed consent form is returned, the individuals’ medical records will be re-accessed to collect information to assess the quality and completeness of any ACP or ACD, and whether there is concordance of care with the individuals expressed wishes, values and beliefs.

#### Post-evaluative survey for and interviews with people with chronic diseases/SDMs at control and intervention sites

Individuals will be invited to participate in a post interview and/or survey. These will be distributed to potential participants either in person (Intervention site) by RN ACP Facilitators (Intervention site) or via post (Control site) by Administrative Officer, who are not the members of the research team.

The Information Statement posted to these participants will emphasise that participation is voluntary, that refusal to participate will not impact upon their current health care services, and that any data collected would be anonymous and be will be stored confidentially.

#### Evaluative survey for and individual/focus group interviews with healthcare professionals

Healthcare professionals (MOs, RNs, SWs & RN ACP Facilitators) at intervention sites will be invited to complete a short survey and post-evaluation interview shortly after the six-month intervention period.

There will be packages containing information statements, interview consent forms, surveys and self-addressed return envelopes available in the tea room, MOs’ office, and nurses’ station of the selected wards/community centres. The decision to participate in survey and/or interview is that of the healthcare professionals and their decision not to participate will not impact upon their employment or future training opportunities. Participant experience for all groups is summarised in Table 4.

**Table 4:** Participant experience for all groups

Groups	Intervention sites			Control sites		
	Intervention	Interview	Survey	Intervention	Interview	Survey
Group 1: People with Chronic Diseases & Group 2: SDMs	✓	✓	✓	✘	✓	✓
Group 3: MOs, RNs & SWs	✓	✓	✓	N/A	N/A	N/A
Group 4: RN ACP facilitators	✓	✓	✓	N/A	N/A	N/A

*SDMs* Substitute Decision Makers, *MO* Medical Officers, *RNs* Registered Nurses, *SW* Social Workers, Advance Care Planning: *ACP*

### Ethical considerations

The study has been approved by the Hunter New England Human Research Ethics Committee, approval no. 17/12/13/4.16 and registered at the Australian New Zealand Clinical Trials Registry, trial ID: ACTRN12618001627246.

There is no actual direct physical, psychological, and economic harm to participants in this study. A number of strategies have been implemented in the study design and processes to ensure autonomy, beneficence, non-maleficence, justice, equity, privacy and confidentiality. The risks and benefits will be clearly stated in information statement that will be distributed to each group of potential participants. Informed consent will be sought and obtained for uptake of ACP service and participation in interviews, and consent will be implied when the survey is returned.

#### Risks

Depending on individuals’ background and circumstances, some participants may experience a degree of discomfort with some aspects of the project. If participation in conversations and interview causes personal distress or discomfort, it will be stopped immediately, and they will be offered support services by appropriate personnel. Participants have the right to withdraw from the project at any time without any disadvantage.

#### Benefits

In the previous work done by the research team, people who shared their experiences in interviews found the interviews therapeutic. Those patients and families who engage in ACP service may experience an increased sense of feeling cared for and understood. Patients’ wishes and preferences for care documented in ACDs will be respected, and families and health professionals are eased from the burden of decision making on patients’ behalf.

The experiences including challenges and enablers that the participants share will be beneficial for researchers and clinicians to understand what it means to dis/engage in ACP service and what needs to be done to improve normalisation of ACP and support services in future.

### Data analysis

#### Qualitative data collection & analysis: audio-recorded individual/focus interviews

Qualitative data will be collected through audits, surveys, individual interviews and/or focus groups for both baseline and post-intervention. Content of Individual/Focus Interviews will be audio recorded (if consent given) and will be transcribed to text. Textual data will be broken down, line-by-line, and open coded i.e. specific categories relevant to the research aim and questions will be pre-identified and labelled but new categories and sub-categories will be added during analysis. Open codes will be systematically inspected and scrutinised in relation to a ‘paradigm model’ in NTP. With the research aims and questions in mind, the analysis will focus on the agents (who), coherence in conditions or context (where, when, why), coherent/collective actions/interactions (process) and reflexive monitoring (e.g. outcomes). NVivo Software will be used to handle the large amount of data.

Textual analysis (directed content analysis) will be undertaken on the following:

- Transcripts of interviews with people with chronic diseases & SDMs
- Transcripts of Individual/Focus Interviews with healthcare professionals (MO, RN, SW, RN ACP Facilitator)
- Completed open-ended questions from Surveys of people with chronic diseases & SDMs, healthcare professionals (MO, RN, SW, RN ACP Facilitator).

Directed content analysis will also be undertaken on information collected/recorded during delivery of the research intervention (facilitated conversations documented in medical notes and on a ‘conversation log’ and potential completion of a ‘conversation card’ and other ACP related documents, as follows:

- Questions, concerns, comments, reasons for uptake and refusal of the service (i.e. reasons for consenting to, or declining participation in the study).
- What participants already know [about ACP], and want to know.
- The extent and the recorded reasons for discordance [between patients’ and SDMs’ expressed wishes and the actual care they received].
- The experiences of patients and SDMs with ACP.
- The experiences of ‘normalisation agents’ [MOs, RNS &/or SWs and RN ACP Facilitators].

The reporting of qualitative measures will follow the Standards for Reporting Qualitative Research (SRQR) [25].

#### Quantitative data analysis

Participant demographics and characteristics will be collected at each site and will be tabulated to show the distribution of participant characteristics between control and intervention sites (i.e. age, sex, primary reason for admission &/or chronic health condition). Continuous variables will be described by mean (standard deviation) if normally distributed and by median (interquartile range) otherwise. Categorical variables will be described by count (percentage). Group comparisons for continuous outcomes will be undertaken by either the Student t-test or Mann-Whitney U test. The Fisher’s Exact test will be used for categorical outcomes.

Baseline characteristics of study groups will be compared to assess similarity at study entry, thereby allowing for the identification of significant imbalances requiring adjustment during analysis. To account this and for clustering at the site level, multi-level regression models with mixed effects will be built to estimate interventional effect. For each model building process, variables found to be significantly associated with respective outcome measures in the univariable analysis at the 10% level will be retained in the final modelling.

As the primary predictor of interest, intervention effect will be forced into the multivariable modelling. Continuous outcome measures will be analysed using linear regression models. For binary outcome measures, analysis will be by binary logistic regression, while multinominal logistic regression will be used to analyse non-binary categorical measures. Model diagnostics will be conducted on all models.

All tests of significance will be set at 5%. Statistical analysis will be performed using Stata version 15.0 (StataCorp, College Station, TX, USA).

### Health economic evaluation

This project will assess the cost-effectiveness of ACP normalisation for people with chronic diseases in acute and community settings within the LHD 1 and LHD 2.

To facilitate this, estimates of intervention effect (i.e. the number of ACDs completed) will be measured at pre and post intervention. Decision tree modelling, populated by effect and cost estimates will then be used to estimate intervention impact in the form of incremental cost-effectiveness ratios (ICERs) and their respective levels of precision. Discounting of costs will not be incorporated into the modelling due to follow-up being less than 1 year. One-way sensitivity analyses will be conducted by varying model inputs within a range representing high and low plausible values. Monte Carlo simulation will be used to assess the robustness of our results by varying all model inputs simultaneously over 10,000 iterations in Ersatz v1.3 (Epigear, 2009).

### Data management

Other than the data collected in the completed ACDs, which should and will be made available to treating healthcare professionals, all data will be non-identifiable. Any personal information from survey and interview will be kept anonymous, and it will be recorded as code and will not be possible to identify participants. Identifying data (i.e. names and contact details) will be stored separately in a password protected file.

All data obtained for this project will be stored in a secure manner. Data from interviews and surveys will be stored electronically in password protected files on the University server. Any data collected in paper copy will be stored separately in locked filing cabinets. Only the key research personnel will have access to this data.

## Discussion

Given the nature of study design, it is not possible to ensure that participants in this study will be representative of the entire population of people with chronic conditions and their SDMs. It is also possible that the exclusion of participants with chronic diseases other than listed may limit the generalisability. It is also important to acknowledge the limitations derived from the nature of self-reported interview and/or survey including response bias and social desirability bias which may lower reliability and validity. The findings from self-reported data will provide valuable insights to answer secondary outcomes measures. The study will be well powered to test the primary outcome measure and the cost-effectiveness calculations.

The benefits of ACP are well known. Patients’ wishes and preferences for care will be respected, and families and health professionals will be eased from the burden of decision-making on patients’ behalf. But more importantly, normalised ACP will promote the actual process of discussing end-of-life issues, leaving patients and families with an increased sense of feeling of cared for and understood. Furthermore, it will assist the uptake of ‘Planning ahead’ practices among people with chronic disease in acute and community settings.

To the Authors knowledge, NPT has not previously been used to develop, implement, and evaluate ACP for people with chronic diseases in acute and community settings. The evidence generated from this project will open a new level of understanding of end-of-life care needs of people and the meanings attached to ACP. It will also contribute to development of new best practice model to normalise ACP across acute and community settings, which is sustainable, transferable and scalable. The normalisation of ACP provides people with chronic diseases and their family, clinicians and policy makers with a feasible and essential opportunity to focus on what matters to people in life, at the end of life, and at the very end of life.

## Abbreviations

ACD: Advanced Care Directive
ACP: Advanced care planning
CC: Central Coast
CCS: Community Control Site
CIS: Community Intervention Site
ED: Emergency department
EOL: End of life
HCS: Hospital Control Site
HIS: Hospital Intervention Site
HNE: Hunter New England
ICU: Intensive care unit
LHD: Local Health District
MMSE: Mini-Mental State Examination
MO: Medical Officer
MOCA: Montreal Cognitive Assessment
MRN: Medical Record Numbers
NPT: Normalisation Process Theory
NSW: New South Wales
RN: Registered Nurse
SDM: Substitute decision-maker
SRQR: Standards for Reporting Qualitative Research
SW: Social Workers
